# Pisa syndrome associated with mirtazapine: a case report

**DOI:** 10.1186/s40360-018-0272-8

**Published:** 2018-12-06

**Authors:** Yuji Yamada, Harumasa Takano, Maki Yamada, Naoko Satake, Naotsugu Hirabayashi, Mitsutoshi Okazaki, Kazuyuki Nakagome

**Affiliations:** 1 Department of Psychiatry, National Center Hospital, National Center of Neurology and Psychiatry, 4-1-1 Ogawahigashi-cho, Kodaira, Tokyo, 187-8551 Japan; 2 Department of Clinical Neuroimaging, Integrative Brain Imaging Center, National Center of Neurology and Psychiatry, Tokyo, Japan; 3 National Institute of Mental Health, National Center of Neurology and Psychiatry, Tokyo, Japan

**Keywords:** Mirtazapine, Side-effects, Dystonia, Pisa syndrome, Bipolar disorder

## Abstract

**Background:**

Mirtazapine is a noradrenergic and specific serotonergic antidepressant; its pharmacological profile indicates a low risk for dopaminergic adverse effects. To date, there has been only a single case report of Pisa syndrome associated with mirtazapine.

**Case presentation:**

The authors report a case involving a 79-year-old woman with bipolar disorder, in whom Pisa syndrome occurred after introduction of mirtazapine, and completely disappeared 3 days after suspension of the drug.

**Conclusions:**

Aspects of this particular case suggest that Pisa syndrome is a possible side effect of Mirtazapine.

## Background

Mirtazapine (MTZ) is a noradrenergic and specific serotonergic antidepressant, which is also classified as a tetracyclic antidepressant [1]. Because MTZ does not strongly block the dopamine D1/D2 receptor, there are few reports describing parkinsonism caused by the drug. In the three clinical trials undertaken in Japan, the incidence of dyskinesia was 0–1.0% but there was no report in terms of parkinsonism or dystonia [2]. In the English literature, there are only two case reports describing dystonia with MTZ use, with one involving Pisa syndrome [3, 4], both of which are cases of major depression. Pisa syndrome is a type of dystonia originally described by Ekbom et al. (1972) [5], which produces abnormally sustained posture with a lateral inclination of the trunk and a degree of backward axial rotation. Drug-induced Pisa syndrome is commonly observed in patients undergoing long-term antipsychotic treatment. However, it has also been reported―albeit less frequently―in patients who receive other medications such as tricyclic antidepressants, selective serotonin reuptake inhibitors, cholinesterase inhibitors, antiemetic drugs, lithium carbonate, benzodiazepines, and tiapride [6]. The present report describes a case of Pisa syndrome that manifested after administration of MTZ to a patient previously diagnosed with bipolar disorder.

## Case report

The patient was a 79-year-old Japanese woman, who provided informed consent for publication of anonymized case details. She had a history of high blood pressure, which had been treated with 80 mg/day valsartan and 5 mg/day amlodipine since the age of 55. No family history of epilepsy or movement disorders was noted. She was diagnosed with bipolar disorder when she was 64 years of age, and treatment was started with 200 mg/day lithium carbonate and 400 mg/day sodium valproate. At least four depressed and five manic phases appeared between ages 64 and 79 years. At 79 years of age, she was admitted to the authors’ hospital with serious depression. Three months before the admission at 79 years of age for severe depression, olanzapine was used to treat mania for 6 weeks with a maximum dose of 12.5 mg/day. Two weeks before the admission, 12.5 mg/day quetiapine was added to treat depression. There were no adverse effects including extrapyramidal symptoms during her previous treatment. Once 15 mg/day MTZ was administered, quetiapine was tapered off within 7 days. Three days after administration of 15 mg/day MTZ, she exhibited symptoms of parkinsonism including short-step gait, rigidity, and tremor. Moreover, 12 days after initiation of MTZ administration, when the dose level had been raised to 22.5 mg/day for 5 days, she presented with an abnormal maintained posture of the trunk, which had a lateral deviation to the right side. No other localizing signs were found, and no changes had been made to her usual medication after the introduction of MTZ. Blood and biochemical screening, and brain computed tomography were normal. Although her cognitive function was preserved (Mini-Mental State Examination score 22/30), the possibility of developing Parkinson’s disease or Lewy body disease was considered. Brain perfusion single-photon emission computed tomography with ^99m^Tc-ethyl cistainate dimer revealed mild decreases in cerebral blood flow in both sides of the frontal lobe. Myocardial metaiodobenzylguanidine (MIBG) scintigraphy revealed no dysfunction in the sympathetic nerves. Dopamine transporter imaging with ^123^I ioflupane did not reveal nigrostriatal degeneration. From the results of these imaging tests, the possibility of idiopathic Parkinson’s disease or Lewy body disease was ruled out. The patient was diagnosed with Pisa syndrome and the MTZ was quickly tapered off; 3 days later, dystonia completely disappeared.

## Discussion

In this case, 12 days after initiation of MTZ administration, when the dose level reached 22.5 mg/day for 5 days, the patient presented with Pisa syndrome; 3 days after suspension of the drug, the syndrome completely disappeared. Furthermore, the results of imaging tests ruled out the possibility of organic disease(s). Accordingly, Pisa syndrome was suspected to have been associated with MTZ.

This is the second report of Pisa syndrome associated with MTZ. Moreover, this is the first case report of Pisa syndrome in which the possibility of idiopathic Parkinson’s disease and Lewy body disease was carefully excluded using MIBG scintigraphy and dopamine transporter imaging with ^123^I ioflupane.

The mechanism through which MTZ produces Pisa syndrome remains unclear. The first possible explanation comes from the fact that MTZ has the largest capacity of all antidepressants to bind to the dopamine D1/D2 receptor [1, 6, 7]. Specifically, the dopamine D1 receptor’s half maximal (50%) inhibitory concentration (IC50) of MTZ is 1600 nM, and the dopamine D2 receptor’s IC50 is 2500 nM. However, antipsychotic drugs, such as quetiapine, which had a much larger binding capacity to the dopamine D1/D2 receptor than MTZ, did not cause Pisa syndrome in this patient. The second possible explanation is that noradrenergic-dopaminergic imbalance produced Pisa syndrome [6, 8]. More precisely, it is known that noradrenaline metabolism is impaired in dystonia, so markedly increased noradrenaline level compared to dopamine level in substantia nigra and red nucleus is found in patients with dystonia [9, 10]. Thus, much higher concentrations of noradrenaline in those areas than those of dopamine associated with MTZ may have produced Pisa syndrome. However, in contrast to this view, it is suggested that MTZ induces the enhancement of the output of dopamine mediated via blockade of α2-adrenergic receptors and facilitation of post-synaptic 5-HT1A function [11]. The last potential explanation is the enhancement of the action of MTZ possibly induced by pharmacokinetic drug-drug interactions. As biotransformation of MTZ is mainly mediated by cytochrome P450(CYP)2D6 and CYP3A4 isoenzymes, inhibitors of these isoenzymes may cause modest increase in MTZ plasma concentrations. However, the adjunctive drugs administered to the patient, such as quetiapine, lithium carbonate, sodium valproate, valsartan and amlodipine, do not affect these isoenzymes [12]. Therefore, drug-drug interactions have a low possibility to be a cause of Pisa syndrome in this patient. In addition, one case report described tardive dystonia induced by the calcium-channel blocker amlodipine [13]. Moreover, a review reported that 5% of drug-induced parkinsonism was caused by calcium-channel blocker [14]. Thus, amlodipine is a potentially suspect drug to induce Pisa syndrome. The review reported that 69% of drug-induced parkinsonism cases developed during the first 3 months after introduction of calcium-channel blockers, with an additional 20% within 12 months [14]. Research has suggested that Pisa syndrome should have occurred within 1 year after drug introduction, if amlodipine is the causative agent. However, the present patient had been using amlodipine for more than 20 years. Therefore, there is little possibility of amlodipine-induced Pisa syndrome in this case.

## Conclusion

We report the second case of Pisa syndrome that occurred after the introduction of MTZ, and completely disappeared 3 days after suspension of the drug. Aspects of this particular case suggest that Pisa syndrome is a possible side effect of MTZ.
